# Gianotti-Crosti Syndrome: A Rare Presentation of the Influenza Virus

**DOI:** 10.7759/cureus.89252

**Published:** 2025-08-02

**Authors:** Ana Sofia Nunes, Lara Navarro, Ana Paula Vieira, Sofia Martins

**Affiliations:** 1 Department of Paediatrics, Hospital de Braga, Braga, PRT; 2 Department of Dermatology, Hospital de Braga, Braga, PRT

**Keywords:** fever with rash, gianotti-crosti syndrome, influenza a virus, influenza virus type a, maculo-papular rash, rash in children

## Abstract

Gianotti-Crosti Syndrome (GCS) is a benign, self-limited dermatologic condition that predominantly affects pediatric patients. It is most commonly associated with viral infections; however, reports implicating Influenza A virus (IAV) as an etiological agent are exceedingly rare.

We report the case of a previously healthy two-year-old girl who presented with fever, rhinorrhea, and a symmetric papulovesicular rash involving the extremities, face, and trunk. Laboratory evaluation revealed leukopenia, neutropenia, and mildly elevated aspartate aminotransferase levels. Serological testing for Epstein-Barr virus (EBV), cytomegalovirus (CMV), and parvovirus B19 was negative, while antigen-based nasal swab testing confirmed the presence of IAV. Based on the characteristic clinical features and exclusion of other common causes, a diagnosis of GCS secondary to IAV infection was established. The patient received symptomatic treatment, and the rash resolved completely within 12 days, with no residual skin changes or complications.

This case highlights a rare but clinically relevant association between IAV and GCS. Although cutaneous manifestations of IAV infection are uncommon, they should be considered in the differential diagnosis of GCS, particularly during influenza season. Prompt recognition of this condition is essential to avoid unnecessary investigations and to provide appropriate guidance and reassurance to caregivers.

## Introduction

Gianotti-Crosti syndrome (GCS), also known as papular acrodermatitis of childhood, is a benign and self-limited dermatologic condition primarily affecting preschool-aged children [1-4]. It is usually preceded by symptoms of a viral infection, followed by a symmetric pink-brown papular or papulovesicular rash that typically exhibits an acral distribution and can affect the face, arms, legs, and buttocks [1, 2, 5]. The extensor surfaces of the extremities are commonly involved, while the trunk is relatively spared [2,4,5,6,7]. Individual lesions range from 1 to 10 millimeters in diameter and may coalesce over pressure points such as the knees and elbows [1, 2, 6]. Mild to moderate pruritus may be present [2-7]. The presence of extracutaneous manifestations, such as fever, malaise, diarrhea, lymphadenopathy, and hepatomegaly, varies between patients [1,2,4,5,6,7]. 

GCS is triggered by multiple viral infections, including Epstein-Barr virus (EBV), cytomegalovirus (CMV), hepatitis A and B virus, enteroviruses, parvovirus, parainfluenza virus, rotavirus, respiratory syncytial virus (RSV), human immunodeficiency virus, and human herpesvirus 6, among others [1,2,5,6,7]. To the best of our knowledge, there is only one documented case of GCS secondary to Influenza A virus (IAV) infection in pediatric patients [3]. The exact mechanism underlying GCS is not fully understood, but it is believed to result from a delayed hypersensitivity reaction to viral infections [1,2,4,6,7]. Furthermore, GCS has been associated with immunizations, including influenza vaccines, as well as bacterial infections [1,2,4,6]. 

A systematic anamnesis and a meticulous physical examination are typically sufficient to diagnose GCS. Laboratory tests are not required to confirm the diagnosis, though serological tests might be needed to identify the underlying viral infection. In challenging cases, such as atypical presentations or in immunocompromised patients, a skin biopsy may be necessary [1,2,4,6,7]. Skin biopsy findings generally include endothelial cell swelling and perivascular inflammatory cell infiltrate [2].

Management of GCS primarily involves symptomatic treatment, including the use of antipyretics, topical emollients, antihistamines, and, in more severe cases, topical or systemic corticosteroids [1,5,6,7]. Spontaneous resolution of the rash typically occurs within two to eight weeks, with no scarring or alterations in skin pigmentation [1,2,5,8]. Most children recover without complications, and recurrences are uncommon. 

The aim of this article is to present a rare case of GCS associated with IAV infection in a previously healthy child, emphasizing the importance of including this uncommon etiological agent in the differential diagnosis, particularly during influenza season.

## Case presentation

During the winter season, a previously healthy two-year-old girl was brought to the emergency department due to a persistent rash that had been worsening over a period of five days. The rash initially appeared on the left underarm and gradually spread symmetrically to her legs, buttocks, face, and trunk. She also had a low-grade fever for two days, rhinorrhea, and mild pruritus. No gastrointestinal symptoms were reported. She was treated in another facility with dimetindene 0.03 mg/kg/day for three days, but the rash did not improve. Her immunizations were up to date, with the last occurring 18 months ago. She was vaccinated for the hepatitis B virus but not for the influenza virus. She lived with her parents in an urban area with no animal exposure or recent travel. There were no cohabitants with the same symptoms. The consumption of suspicious food and the use of new hygiene products or clothes were ruled out.

**Figure 1:**
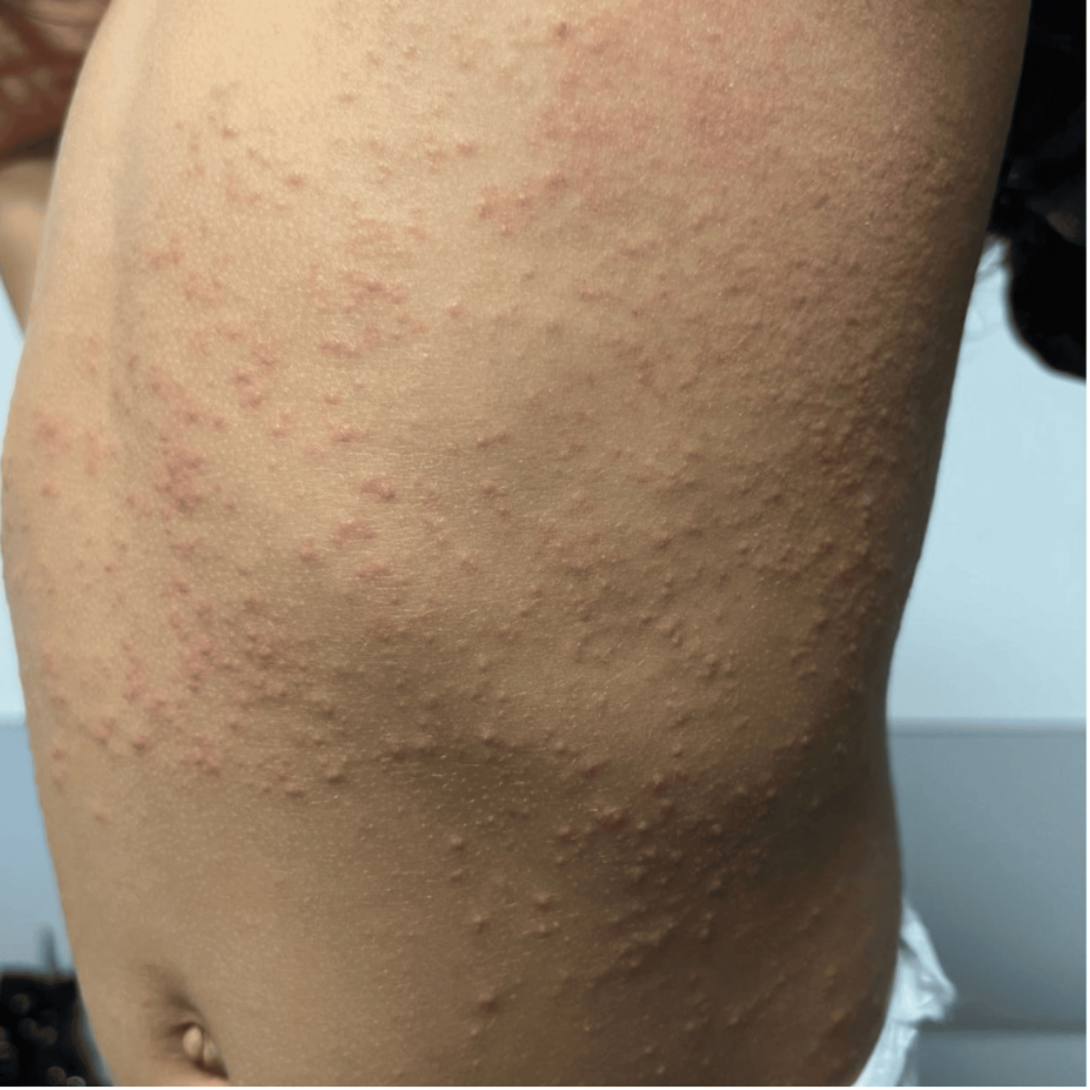
Papular rash on the trunk compatible with Gianotti-Crosti syndrome

**Figure 2:**
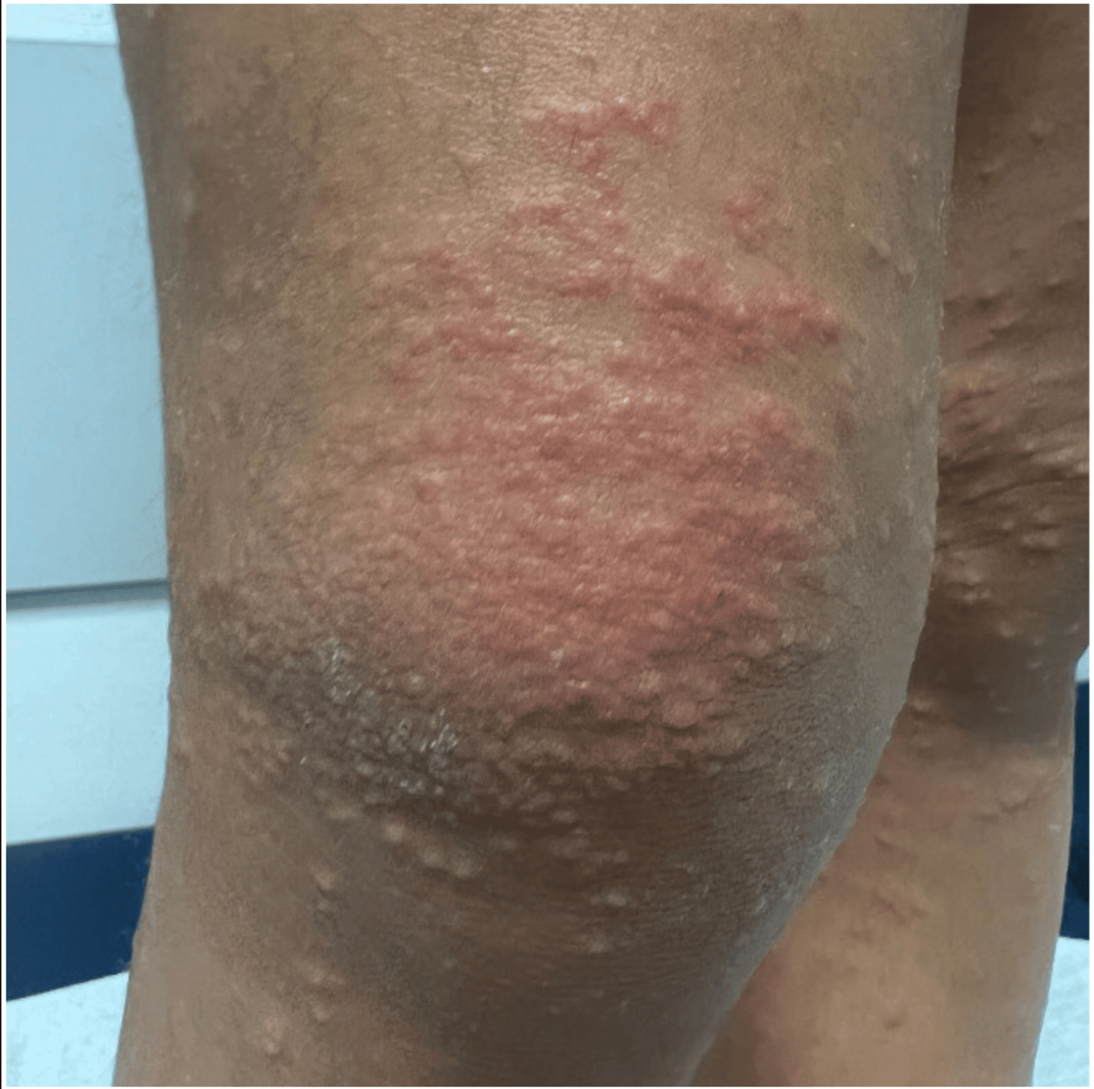
Papular rash compatible with Gianotti-Crosti syndrome, confluent over the knee

On physical examination, she presented with an axillary temperature of 36ºC, blood pressure of 97/62 mmHg, heart rate of 93 beats per minute, respiratory rate of 23 breaths per minute, and transcutaneous oxygen saturation of 99% in ambient air. Additionally, a symmetrical pink papulovesicular rash was observed, most prominent on her knees, arms, legs, buttocks, left anterior aspect of the chest, and cheeks, but sparing her palms and soles (Figures 1, 2). Petechiae, purpura, bruises, or ecchymosis were not identified. The oropharyngeal examination was normal. Hepatomegaly and jaundice were not observed. There were no palpable lymph nodes or peripheral edema. The remaining physical examination did not reveal any noteworthy findings.

Further investigation showed a normal hemoglobin, a low white blood cell count, a low neutrophil count, a slight increase in aspartate aminotransferase but normal alanine transaminase, and a low C-reactive protein (Table 1). Serological tests for EBV, CMV, and parvovirus B19 were negative. The detection of type 1 and 2 herpes simplex virus DNA yielded negative results. An antigen-based nasal swab tested positive for IAV but negative for influenza B virus, parainfluenza 1, 2, and 3, RSV, adenovirus, metapneumovirus, severe acute respiratory syndrome coronavirus 2 (SARS‑CoV‑2), and human coronavirus OC43 (Table 1).

**Table 1:** List of complementary diagnostic tests

TEST	RESULT	REFERENCE RANGE
COMPLETE BLOOD COUNT		
Hemoglobin	12.4	10.5 – 13.5 g/dL
Hematocrit	35.2	33 – 39 %
Leukocytes	3500	6000 – 17000 /uL
Neutrophils	1000	1500 – 8500 /uL
Lymphocytes	2200	3000 – 9500 /uL
Platelets	182000	> 150000 /uL
Erythrocyte sedimentation rate	6	1 – 20 mm/h
BLOOD CHEMISTRY		
Glucose	82	74 – 106 mg/dL
Urea	22	19 – 49 mg/dL
Creatinine	0.3	0.24 – 0.41 mg/dL
Sodium	139	135 – 145 mmol/L
Potassium	4.3	3.5 – 5.1 mmol/L
Chloride	107	98 – 107 mmol/L
Aspartate aminotransferase (AST)	53	< 50 U/L
Alanine transaminase (ALT)	20	7 – 40 U/L
C-Reactive protein	< 0.5	< 5 mg/L
MICROBIOLOGY		
Hemoculture	Negative	-
DNA QUANTIFICATION (POLYMERASE CHAIN REACTION)		
Herpes simplex I and II	Negative	-
SEROLOGIES		
IgG Cytomegalovirus (CMV)	Negative (0.198 index)	Negative: < 0.9
		Equivocal: 0.9-1.1
		Positive: > 1.1
IgM CMV	Negative (0.239 index)	Negative: < 0.9
		Equivocal: 0.9-1.1
		Positive: > 1.1
IgG Epstein-Barr virus (EBV) early antigen	Negative (<< 5.00)	Negative: < 5 U/mL
		Equivocal: 5–10 U/mL
		Positive: > 10 U/mL
IgG EBV viral capsid antigen	Positive (59.2)	Negative: < 20 U/mL
		Equivocal: 20–40 U/mL
		Positive: > 40 U/mL
IgM EBV viral capsid antigen	Negative (14.3)	Negative: < 20 U/mL
		Equivocal: 20–40 U/mL
		Positive: > 40 U/mL
IgG EBV nuclear antigen	Positive (165)	Negative: < 5 U/mL
		Equivocal: 5–20 U/mL
		Positive: > 20 U/mL
Monospot	Negative	-
IgG Parvovirus B19	Negative (<< 0.10 index)	Negative: < 0.9
		Doubtful: 1 - 1.1
		Positive: > 1.1
IgM Parvovirus B19	Negative (<< 0.10 index)	Negative: < 0.9
		Doubtful: 1 - 1.1
		Positive: > 1.1
ANTIGEN-BASED NASAL SWAB		
Respiratory syncytial virus	Negative	-
Influenza A	Positive	-
Influenza B	Negative	-
Parainfluenza 1	Negative	-
Parainfluenza 2	Negative	-
Parainfluenza 3	Negative	-
Adenovirus	Negative	-
Metapneumovirus	Negative	-
SARS-CoV-2	Negative	-
Coronavirus OC43	Negative	-

Based on the clinical presentation, the characteristics of the rash, and the identification of a viral agent, a diagnosis of GCS was made. Given the toddler’s good general condition, she was discharged with oral hydroxyzine 2 mg/kg/day, and the typical evolution of GCS was explained to the family. Four days after evaluation at the emergency department, she was also evaluated by a dermatologist who clinically confirmed the diagnosis and did not prescribe any additional medication. The rash persisted for 12 days and resolved without scarring, hypopigmentation, or hyperpigmentation. Two weeks later, she was re-evaluated during a pediatric consultation, where a complete resolution of the exanthema was noted. A complete blood count showed normalized blood parameters.

## Discussion

GCS is a well-documented pediatric dermatosis that typically follows viral infections [1-7]. Although it is classically associated with EBV, hepatitis B virus, and CMV, the spectrum of implicated pathogens has expanded to include a variety of viral and, less frequently, bacterial agents [1,2,5,6,7]. Nonetheless, IAV has only been reported as a rare causative pathogen. To our knowledge, this is one of the very few documented pediatric cases of GCS attributed to IAV infection [3].

While the pathogenesis of GCS remains incompletely understood, it is widely believed to represent a virus-triggered immunologic phenomenon, possibly mediated by a delayed-type hypersensitivity reaction [1,2,4,6,7]. The skin findings, typically self-limited and non-scarring, result from a perivascular inflammatory infiltrate, as documented in histopathologic studies [2]. The immunologic response to viral antigens, whether due to direct viral involvement, molecular mimicry, or immune complex deposition, could explain the dermatologic presentation. This proposed mechanism may also account for the rare association between IAV and GCS, as influenza viruses are not typically associated with cutaneous tropism [9, 10].

In this report, we describe a clinically typical case of GCS in a previously healthy two-year-old girl during the peak influenza season. The patient presented with a symmetric papulovesicular eruption affecting the face, extremities, and buttocks. Involvement of the trunk is less frequent; however, it does not preclude the diagnosis of GCS [2]. The exclusion of alternative diagnoses and the confirmation of IAV through antigen testing strongly support a causal link between IAV and the observed dermatologic presentation.

The present case further illustrates the importance of including IAV in the differential diagnosis of GCS, especially in the context of seasonal epidemics. Diagnosis of GCS is primarily clinical and supported by a thorough history and physical examination. In this case, the diagnosis was established without the need for a skin biopsy, highlighting the value of clinical expertise in pediatric dermatology. Laboratory findings, including transient leukopenia and mild transaminase elevation, have been previously reported in GCS and may reflect a mild systemic inflammatory response to viral infection. Management of GCS is supportive and includes topical emollients, antihistamines for pruritus, and reassurance to the caregivers [1,5,6,7]. As seen in our patient, the rash resolved completely within two weeks, without recurrence or sequelae. Follow-up confirmed normalization of laboratory parameters and full dermatologic recovery, affirming the self-limited nature of the condition.

This clinical case highlights an uncommon etiological agent responsible for GCS. The detection of IAV in nasopharyngeal secretions through direct enzyme immunoassay during the peak period of IAV season supports the diagnosis. Therefore, we suggest that IAV should always be considered in the etiological investigation of GCS, despite the infrequent occurrence of cutaneous manifestations resulting from IAV infections. Furthermore, prompt diagnosis of GCS is important to avoid subjecting children to unnecessary tests, to support appropriate outpatient management, and to reassure the family, who are usually concerned about the persistence of the rash.

## Conclusions

GCS is a self-limited dermatologic condition with a well-established association with viral infections. This case highlights a rare but significant link between IAV and GCS, expanding the known spectrum of potential viral triggers. Although cutaneous manifestations of IAV are uncommon, clinicians should consider this virus in the differential diagnosis of GCS, particularly during influenza season. Prompt clinical recognition based on characteristic features can prevent unnecessary diagnostic procedures and facilitate appropriate reassurance and management for both patients and caregivers.
